# Gender-associated role in patients with schizophrenia. Is there a connection with the resistance?

**DOI:** 10.3389/fpsyt.2022.995455

**Published:** 2022-08-10

**Authors:** Georgi Panov

**Affiliations:** Psychiatric Clinic, Trakia University, Stara Zagora, Bulgaria

**Keywords:** resistant schizophrenia, clinical remission, gender identity, sex, gender, social activity, sex role

## Abstract

Schizophrenia is a chronic mental illness observed with equal prevalence in different cultures and ethnic groups. The clinical picture relates to behavior and social adaptation. A significant percentage of patients, despite the implementation of various therapeutic interventions, remain resistant to the ongoing treatment. Occupying a certain gender role depends both on biological belonging and on the way of self-perception characteristic of the given person. Self-perception reflects gender identification, which in social aspect is determined by the choice of social activities performed. Changes in behavior and social adaptation in patients with schizophrenia led us to conduct a study to analyze the perceived gender role in patients with schizophrenia, looking for differences between patients with treatment resistance and those in clinical remission.

**Materials and methods:**

A total of 105 patients with schizophrenia were analyzed. Of them, 45 were with resistant symptoms and 60 in clinical remission. The clinical analysis of the patients was carried out using the PANSS and BPRS scales. The evaluation of the choice of social activity related to a particular gender was done with the Bem Sex-Role Inventory (BSRI).

**Results:**

Out of all 105 patients with schizophrenia, in 80/76.19%/we found a higher identification with the female role, 17/16.19%/made an association with the male role and in 8/7.61%/patients we found the same results, i.e., with both the male and female roles. Among the patients with treatment resistant schizophrenia (TRS)–45, 34/75.56%/identified more with the female gender role, 6/13.33/perceived the male gender role as active, and in 5/11.11%/the identification was equal both with the male and with the female roles. Among the patients in clinical remission (CR)–60, 46/76.67%/accepted the female role as active, 11/18.33/identified with the male one, and three/5%/accepted both roles equally. When assessing the relationship between biological sex and perceived gender role, it was found that among men/a total of 39/half identified with the female gender role and half with the male gender role. Among women/a total of 66/, 90% perceived the female gender role, 7%–the male and 3% equally the male and the female gender role. No relationship was found between the choice of a certain gender role and the onset of psychosis and its duration in the observed patients.

**Conclusion:**

We found a higher percentage of schizophrenic patients who showed higher identification with the female gender role. Approximately half of the males identified with the female gender role. Resistance had no influence on the choice of sex-associated social activity. Factors related to the course of the schizophrenia process such as age of onset of psychosis and duration of psychosis was not associated with an influence on identification with sex-associated social activity. Our research suggests that identification with a particular sex associative social activity is most likely established earlier in the prodromal period.

## Introduction

Schizophrenia is a severe chronic mental illness characterized by specific symptoms divided into three large groups: positive, negative and cognitive symptoms (1). Research in recent years has shown that schizophrenia is not just a mental disorder in the narrow sense of the word. It has multiple disturbances in metabolism, opioid system and immunity, which often require further evaluation and therapeutic approach (2–4). In patients with schizophrenia and associative connections as well as loss of connectivity between individual brains regions were found (5–7). The established changes in the connecting systems between the different brain regions give grounds for some authors to consider schizophrenia as a disconnection syndrome (8). On the other hand, it should be noted that gender differences in brain size have been found, as well as not very convincing differences in the associative connections between individual neuronal centers and functional lateralization in men and women (9). Other authors have looked at morphological differences in different brain regions in males and females, concluding that sex-associated biological differences were observed (10).

The course of the schizophrenic process in men and in women is characterized by certain differences related to both the time of appearance and the prevalence of certain symptoms to the higher rate of resistance found in men (11, 12) Numerous studies have been conducted regarding the gender characteristics of patients with schizophrenia, and the gender differences considered were based on the established dichotomy of “male” and “female” gender (13). Authors define gender role as “all those things that one says or does to reveal oneself as having the status of boy or man, girl, or woman.” (14).

In general, communities based on the biological differences between men and women create a set of social expectations that define the behavior that is “appropriate” for men and women (15). Many cultures have different requirements and norms based on gender differences and there is no established universal standard for a male or female role (16, 17) is a cognitive theory that attempts to explain how individuals define their gender in society and how the associated characteristics are maintained and transmitted to other members of the culture (17). The greatest Contribution To The Field of certain social behavior was the attempt to quantify it through the Bem Sex-Role scale. Originally developed as a tool to identify sex-typed individuals, many researchers use it to assess other components of gender, including type of social activity as a measure of masculinity/femininity (18).

Deaux (19) suggests that gender comparison is based on culturally accepted norms regarding the nature of femininity and masculinity. Lewine (20) found it appropriate to use this terminology also in the study of schizophrenic patients. Furthermore, it does not consider how psychological gender, which is primarily a matter of self-perception, may influence illness in men and women with schizophrenia and how this may impact treatment and the recovery process (21). Cultural expectations for men and women with schizophrenia differ according to research. Research has shown that males with schizophrenia have more difficulty performing expected normative role activities than females (21). An analysis of the literature on gender identity and schizophrenia shows that men and women with schizophrenia have disordered gender role identification and this is more characteristic of males, and this fact should be taken into account in the therapeutic approach to patients (22).

An analysis of the literature on gender identity and schizophrenia shows that men and women with schizophrenia have disordered gender role identification and this is more characteristic of males, and this fact should be taken into account in the therapeutic approach to patients (22).

Seeman (23) noted that, based on the major differences between men and women with schizophrenia: from the different age of onset to the different symptomatology, dose, and affective fluctuations, the two sexes cannot be burdened with the same demands in the presence of a schizophrenic process. Authors have found that patients more easily perceive and identify with the female role than with the male role (24). Many psychotic symptoms are found in patients with identity disorder, and in patients with schizophrenia there is a large percentage of patients with identity disorder (25).

Other authors consider identity disorder and schizophrenia as mutually exclusive, raising the question of exaggerating the relationship between schizophrenia and identity disorder (26). Research has shown fluidity in the timing of gender identity expression, which may fluctuate over time and be associated with thought process disorder (27).

Analysis of the effect of treatment in patients with schizophrenia shows that up to 40% are resistant to treatment (28). Schizophrenia is a complex disorder that can be likened to a “symphonic” orchestration, but as other authors have also found, gender is also a consequence of a complex “symphonic” orchestration (29). Naturally, the question arises whether there are differences in gender identification in patients with resistance to treatment and those who have achieved clinical remission.

## Materials and methods

### Subjects

A total of 105 patients with schizophrenia with consecutive psychotic episodes have been observed. Of these, 45 have resistant schizophrenia and the remaining 60 are in clinical remission.

The patients were observed in a psychiatric clinic at the University Hospital in Stara Zagora.

Including criteria to all patients:

1. Diagnosis of schizophrenia according to the Diagnostic and Statistical Manual of Mental Disorders, Fifth Edition [DSM-V, 2013];
2. Between 18 and 60 years of age;
3. At least primary education;

Including criteria for patients with resistant schizophrenia are those who have met the resistance criteria of the published consensus on resistant schizophrenia (28). These are:

1. Assessment of symptoms with the PANSS and BPRS scale (30, 31).
2. Prospective monitoring for a period of at least 12°weeks.
3. Administration of at least two antipsychotic medication trials at a dose corresponding to or greater than 600 mg chlorpromazine equivalents.
4. Reduction of symptoms when assessed with the PANSS and BPRS scale by less than 20% for the observed period of time.
5. The assessment of social dysfunction using the SOFAS (Social and Occupational Functioning Assessment Scale) scale is below 60.

The exclusion criteria are:

1. Intellectual disability.
2. Psychoactive substance abuse.
2. Presence of organic brain damage.
3. Concomitant progressive neurological or severe somatic diseases.
4. Expressed personality change.
5. Score of MMSE (Mini-Mental State Exam) below 25 points.
6. Pregnancy and breastfeeding.

### Methods

Sandra Bem’s scale (BSRI) was used to assess the perceived gender role (17).

We used the SPSS (version 26) statistical package. Correlation analysis was used to investigate the relationship between the sex-associated role and the resistance to therapy, age of onset of schizophrenia and duration of the disorder in patients with schizophrenia. A non-parametric statistical method was also used (32).

Age, body mass index (BMI), level of education is controlled as covariables.

All research procedures were carried out in accordance with the Declaration of Helsinki.

All patients signed an informed consent before admission to the clinical settings and performing diagnostic tests and therapy.

## Results

The mean age of patients in the group of resistant schizophrenia was 36.98 years. The minimum age is 21 years and the maximum is 60 years.

The mean age of patients in the group of schizophrenia in clinical remission was 37.25 years. The minimum is 23 years and the maximum is 63 years.

We did not find a difference in the mean age of the patients in both groups at the time of the study.

No statistical differences were found between the two groups of patients in terms of height, weight, and BMI.

An analysis of patients with resistant psychosis and those in remission using the Bem Sex-Role Inventory (BSRI) found the following results:

Of all patients with schizophrenia, 80/76.19%/showed a higher scale when measuring identification with the female role, 17/16.19%/made an association with the male role, and 8/7.61%/patients had the same results in identifying with gender roles, i.e., with both the male and female roles.

**FIGURE 1:**
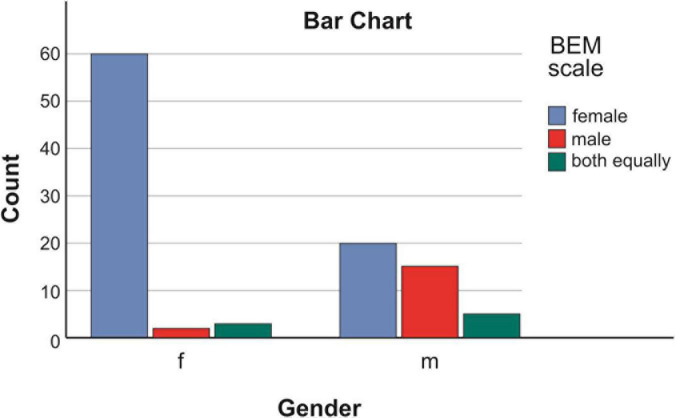
Connection between biological sex and accepted gender role I patients with schizophrenia.

When conducting a comparative study to compare the real sex of the examined patients with the results of using the Sandra Bem scale, the following data were observed:

Among the 65 female persons, it was found that 60/92.31%/identified with the female gender role, 2/3.07%/with the male gender role, and 3/4.62%/perceived both gender roles equally.
Among the 40 males, it was found that 20/50%/identified more with the female gender role, 15/37.5%/identified with the male gender role, and 5/12.5%/perceived both roles equally Figure 1.

These data show that the recorded predominance of female gender role identification is at the expense of males, with half of them identifying with the female gender role.

**FIGURE 2:**
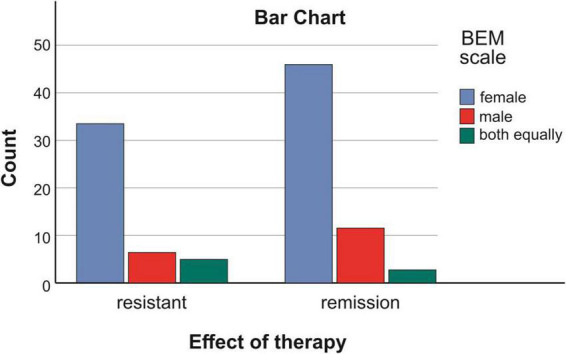
Connection between effect of therapy and accepted gender role in patients with schizophrenia.

The analysis of the relationship between perceived gender role and treatment effectiveness showed the following results:

Among the patients with treatment resistant schizophrenia (TRS)–45, 34/75.56%/identified more with the female gender role, 6/13.33/perceived the male gender role as active, and in 5/11.11%/the identification was equal both with the male and the female roles.
Among the patients in clinical remission (CR)–60, 46/76.67%/accepted the female role as active, 11/18.33/identified with the male one, and three/5%/accepted both roles equally Figure 2 and Table 1.

**TABLE 1:** Connection between effect of therapy and accepted gender role in patients with schizophrenia.

		BEM scale			Total
		female	male	both equally	
Effect of therapy	resistant	34	6	5	45
	remission	46	11	3	60
Total		80	17	8	105

With this observation of ours in schizophrenic patients, we found that easy identification and acceptance of the female gender role was observed.
In view of the described and assumed factors related to the change in the gender role, we made the following analyses of the relationship with the perceived specific gender role. We analyzed the relationship between the accepted gender role and the age; the age of onset of the schizophrenia process as well as the duration of the schizophrenia process.

When analyzing the relationship between the perceived gender role as male, female, and equally both female and male with the age of the patients, we found the following:

The average age of patients who adopted the female gender role was 37.44 years.
The average age of patients who identified with the male gender role was 32 years.
The average age of those who had the same results in both male and male attitudes was 44 years.
The conducted statistical analyses showed no statistically significant relationship: *p* < 0.05.

Assessment of the relationship between the onset of schizophrenia and perceived gender role showed the following results:

Among those with a perceived female role, the onset of schizophrenia was at 26.23 years. For those identified with the male gender role–23.94 years and for those with identification equally with both roles–21.88 years. Statistical analysis showed no significant relationship *p* < 0.05.

The analysis of the relationship between the accepted gender role and the duration of the schizophrenia process showed the following distribution:

In patients who accepted the female gender role, the duration was 11.25 years. For those with a male perception, it was 9.18 years, and for those who identified with both gender roles–22.5 years. Despite the differences in the length of years, the sample of patients with the same points regarding the evaluation of male and female gender roles was small–eight patients Table 2.

**TABLE 2:** Relationship between features of psychosis and perceived gender role.

	Female	Male	Both equally	Sign.
Age	37.44	32	44	*p* > 0.05
Age of onset	26.23	23.94	21.88	*p* > 0.05
Duration of Sch	11.5	9.18	22.5	*p* > 0.05

The conducted statistical analysis showed no statistically significant difference with *p* < 0.05.

## Discussion

The results of the study conducted to assess the identity with a certain role in patients with showed that two thirds of the observed patients identified with the female role. Among the male subjects, half of the patients identified more with the female role. Just under a fifth identified as masculine and just under a tenth scored equally on masculine and feminine gender roles. Our observation showed that it was done by easy identification and acceptance of the female role. These data confirm the observations of other authors that there is a change in gender identification in patients with schizophrenia (21, 22, 24, 25).

There were almost identical results regarding the discrepancy between biological sex and the points indicating dominant gender roles in both resistant and remitted schizophrenic patients. These results showed that this registered change was more deeply affecting the identity of the individual and is not only related to the loss of the patients’ social role and autonomy.

The view of some authors (29) that mental illness itself can be considered as silencing other factors of identity is difficult to accept due to the fact that in patients in remission a relatively preserved or largely recovered level of functional activity is observed, despite changing their gender identification. On the other hand, we should not forget the fact that gender places higher demands on men in relation to the need for professional and social connections. These factors, on the other hand, are also a noted problem in schizophrenics in general (33). Seen in this way, some explanation can be given to this “escape” from the male role. We did not find confirmation of the hypothesis that most likely the early onset of the disease and its duration make it difficult to construct sex-associated patterns (33). Our data showed no association between the onset of psychosis, its duration, and gender role identification. We believe that most likely the choice of the female role and identification with it is related to being placed in a subordinate, “protected” position in patients with schizophrenia, on the one hand, and on the other hand, these data can be interpreted through the prism of “burden of normality” (34).

In these cases, a bridge can definitely be drawn between the choice of gender role and the phenomenon of “learned helplessness”, which includes cognitive, motivational and emotional disorders appearing as a consequence of the impact of uncontrollable negative life events (35) and places patients in a more “subordinate position”. These results of ours indirectly support the idea of a relationship between identity disorder and schizophrenia, not supporting those who believe that such a relationship is absent (26).

The result of the lack of relationship between symptom resistance and perceived gender role is interesting. As a possible explanation, we found that with the onset of psychosis a defensive position within the female gender role was adopted, which, despite recovery from psychosis, was more convenient for patients to abandon. On the other hand, patients with schizophrenia often have prodromal symptoms, sometimes years before the development of the disease, which enable the adoption of social strategies within the female gender role, as a more protected social position. We believe that the development of this “escape” from the male gender role began even before the onset of psychotic experiences.

One of the main unanswered questions is whether the accepted gender role will remain stable over time or whether there will be dynamics and change in social patterns of behavior. If we proceed from the concept of the fluidity of gender identity, such a process should also be assumed (27, 36). For this, it is necessary to conduct additional longitudinal studies in patients with schizophrenia in order to track the presence of possible dynamics over time.

At this stage, we cannot give an answer to the question whether in the course of social recovery in patients in clinical remission, a gradient shift of the gender role will not be observed in parallel with the re-adaptation processes.

## Conclusion

We found a change in gender role choice that is characteristic of male individuals. We did not find a relationship between the choice of gender role and resistance to therapy, nor with the features of the schizophrenic process. These data of ours indicate that the change in the dominant gender role most likely occurred even before the onset of schizophrenia, most likely in the prodromal period.

Our observations are a good direction for work in the process of rehabilitation and recovery for patients with schizophrenia. In the course of this process, we consider it important to pay attention to the assessment of the dominant gender role in order to provide more correct strategies for social rehabilitation.

## Data availability statement

The raw data supporting the conclusions of this article will be made available by the authors, without undue reservation.

## Ethics statement

The studies involving human participants were reviewed and approved by Ethical Committee of University Hospital “Stoyan Kirkovich”. The patients/participants provided their written informed consent to participate in this study. The study was conducted in accordance with the Declaration of Helsinki, and approved by the Ethical Committee of University Hospital “Stoyan Kirkovich” Stara Zagora, protocol code TR3-02-242/30 December 2021.

## Author contributions

The author confirms being the sole contributor of this work and has approved it for publication.

## References

[1] Jablensky A. The diagnostic concept of schizophrenia: Its history, evolution, and future prospects. *Dialogues Clin Neurosci.* (2010) 12:271–87. 10.31887/DCNS.2010.12.3/ajablensky 20954425 PMC3181977

[2] Tanaka M Tóth F Polyák H Szabó Á Mándi Y Vécsei L. Immune influencers in action: Metabolites and enzymes of the tryptophan-kynurenine metabolic pathway. *Biomedicines.* (2021) 9:734. 10.3390/biomedicines9070734 34202246 PMC8301407

[3] Correia BSB Nani JV Waladares Ricardo R Stanisic D Costa TBBC Hayashi MAF Effects of psychostimulants and antipsychotics on serum lipids in an animal model for schizophrenia. *Biomedicines.* (2021) 9:235. 10.3390/biomedicines9030235 33652776 PMC7996855

[4] Khandaker GM Cousins L Deakin J Lennox BR Yolken R Jones PB. Inflammation and immunity in schizophrenia: Implications for pathophysiology and treatment. *Lancet Psychiatry.* (2015) 2:258–70. 10.1016/S2215-0366(14)00122-9 26359903 PMC4595998

[5] Stoyanov D Kandilarova S Borgwardt S Stieglitz RD Hugdahl K Kostianev S. Psychopathology assessment methods revisited: On translational cross-validation of clinical self-evaluation scale and fMRI. *Front Psychiatry.* (2018) 9:21. 10.3389/fpsyt.2018.00021 29472876 PMC5809475

[6] Stoyanov D Aryutova K Kandilarova S Paunova R Arabadzhiev Z Todeva-Radneva A Diagnostic task specific activations in functional MRI and aberrant connectivity of insula with middle frontal gyrus can inform the differential diagnosis of psychosis. *Diagnostics.* (2021) 11:95. 10.3390/diagnostics11010095 33435624 PMC7827259

[7] Nyatega CO Qiang L Adamu MJ Younis A Kawuwa HB. Altered dynamic functional connectivity of cuneus in schizophrenia patients: A resting-state fMRI study. *Appl Sci.* (2021) 23:11392. 10.3390/app112311392

[8] Friston K Brown HR Siemerkus J Stephan KE. The dysconnection hypothesis. *Schizophr Res.* (2016) 176:83–94. 10.1016/j.schres.2016.07.014 27450778 PMC5147460

[9] Eliot L Ahmed A Khan H Patel J. Dump the “dimorphism”: Comprehensive synthesis of human brain studies reveals few male-female differences beyond size. *Neurosci Biobehav Rev.* (2021) 125:667–97. 10.1016/j.neubiorev.2021.02.026 33621637

[10] Xin J Zhang Y Tang Y Yang Y. Brain differences between men and women: Evidence from deep learning. *Front Neurosci.* (2019) 13:185. 10.3389/fnins.2019.00185 30906246 PMC6418873

[11] Falkenburg J Tracy DK. Sex and schizophrenia: A review of gender differences. *Psychosis.* (2014) 6:61–9. 10.1080/17522439.2012.733405

[12] Panov G Djulgerova S Panova P. The effect of education level and sex differences on resistance to treatment in patients with schizophrenia. *Bulg Med.* (2022) 1:12.

[13] Kulkarni J. Women and schizophrenia: A review. *Aust N Z J Psychiatry.* (1997) 31:46–56. 10.3109/00048679709073798 9088485

[14] Money J Hampson JG Hampson JL. An examination of some basic sexual concepts: The evidence of human hermaphroditism. *Bull Johns Hopkins Hosp.* (1955) 97:301–19. 13260820

[15] Galdas PM Johnson JL Percy ME Ratner PA. Help seeking for cardiac symptoms: Beyond the masculine–feminine binary. *Soc Sci Med.* (2010) 71:18–24. 10.1016/j.socscimed.2010.03.006 20398989 PMC5142841

[16] Spade J Valentine C. *The kaleidoscope of gender: Prisms, patterns, and possibilities.* 3rd ed. Thousand Oaks, CA: Pine Forge Press (2011).

[17] Bem SL. The measurement of psychological androgyny. *Consult Clin Psychol.* (1974) 42:155–62. 4823550

[18] Hoffman RM Borders LD. Twenty-five years after the Bem sex –role inventory: A reassessment and new issues regarding classification variability. *Meas Eval Couns Dev.* (2001) 34:39–55.

[19] Deaux K. Sorry, wrong number a reply to gentile’s call. *Psychol Sci.* (1993) 4:125–6. 10.1111/j.1467-9280.1993.tb00474.x

[20] Lewine RR. Sex: An imperfect marker of gender. *Schizophr Bull.* (1994) 20:777–9. 10.1093/schbul/20.4.777 7701282

[21] Nasser EH Walders N Jenkins JH. The experience of schizophrenia: What’s gender got to do with it? A critical review of the current status of research on schizophrenia. *Schizophr Bull.* (2002) 28:351–62. 10.1093/oxfordjournals.schbul.a006944 12693440

[22] Strkalj Iveziæ S John N. Gender and schizophrenia. *Psychiatr Danub.* (2009) 21:106–10. 19789493

[23] Seeman MV. Schizophrenic men and women require different treatment programs. *J Psychiatr Treat Eval.* (1983) 5:143–8.

[24] Sajatovic M Jenkins JH Strauss ME Butt ZA Carpenter E. Gender identity and implications for recovery among men and women with schizophrenia. *Psychiatr Serv.* (2005) 56:96–8. 10.1176/appi.ps.56.1.96 15637200

[25] Rajkumar RP. Gender identity disorder and schizophrenia: Neurodevelopmental disorders with common causal mechanisms? *Schizophr Res Treatment.* (2014) 2014:463757. 10.1155/2014/463757 25548672 PMC4274821

[26] Hoshiai M Matsumoto Y Sato T Ohnishi M Okabe N Kishimoto Y Psychiatric comorbidity among patients with gender identity disorder. *Psychiatry Clin Neurosci.* (2010) 64:514–9. 10.1111/j.1440-1819.2010.02118.x 20727112

[27] Gerken AT McGahee S Keuroghlian AS Freudenreich O. Consideration of clozapine and gender-affirming medical care for an HIV-positive person with schizophrenia and fluctuating gender identity. *Harv Rev Psychiatry.* (2016) 24:406–15. 10.1097/HRP.0000000000000120 27824636

[28] Howes OD McCutcheon R Agid O de Bartolomeis A van Beveren NJ Birnbaum ML Treatment-resistant schizophrenia: Treatment response and resistance in psychosis (TRRIP) working group consensus guidelines on diagnosis and terminology. *Am J Psychiatry.* (2017) 174:216–29. 10.1176/appi.ajp.2016.16050503 27919182 PMC6231547

[29] LaTorre RA. The psychological assessment of gender identity and gender role in schizophrenia. *Schizophr Bull.* (1976) 2:266–85. 10.1093/schbul/2.2.266 792990

[30] Overall JE Gorham DR. The brief psychiatric rating scale. *Psychol Rep.* (1962) 10:799–812. 10.2466/pr0.1962.10.3.799

[31] Kay SR Fiszbein A Opler LA. The positive and negative syndrome scale (PANSS) for schizophrenia. *Schizophr Bull.* (1987) 13:261–76. 10.1093/schbul/13.2.261 3616518

[32] Mann HB Whitney DR. On a test of whether one of two random variables is stochastically larger than the other. *Ann Math Stat.* (1947) 18:50–60. 10.1214/aoms/1177730491

[33] Notman MT Nadelson CC. *Women and men.* Washington, DC: American Psychiatric Press (1991).

[34] Gilbert F. The burden of normality: From ‘chronically ill’ to ‘symptom free’. New ethical challenges for deep brain stimulation postoperative treatment. *J Med Ethics.* (2012) 38:408–12. 10.1136/medethics-2011-100044 22431560

[35] Seligman MEP. *Helplessness: On depression, development, and death.* New York, NY: W H Freeman/Times Books/Henry Holt & Co (1975).

[36] Katz-Wise SL. Sexual fluidity in young adult women and men: Associations with sexual orientation and sexual identity development. *Psychol Sex.* (2015) 6:189–208. 10.1080/19419899.2013.876445

